# In This Issue

**DOI:** 10.1002/cfg.126

**Published:** 2001-12


**Comparative and Functional Genomics**
*Comp Funct Genom* 2001; **2**: 353–354.
**DOI:** 10.1002/cfg.126

### In this issue

### Upstream – news in genomics

In this issue we launch our news feature, Upstream. We aim to provide brief reports on prominent papers and press releases in the field of genomics, from complete genome sequences, to announcements from large-scale funding programmes.

### Protein interaction studies combining surface plasmon resonance and mass spectrometry

Mattei *et al.* describe an approach in which surface plasmon resonance was used to capture peptides from the *Fusarium moniliforme* endopolygalacturonase (PG) that interact with the polygalacturonase-inhibiting protein of *Phaseolus vulgaris*, which were then identified by mass spectrometry. This data was used to map the domain of the PG protein that forms the interaction surface. The system was also used to study the effects of PG glycosylation on the interaction between the two proteins.

### What is 'The Grid' and what does it offer to biologists?

Carole Goble describes 'The Grid', a proposed distributed computing infrastructure, and explains what its purpose is, and what it has to offer biologists. Biologists are already working in a manner that the Grid is intended to support, that is, working in large, distributed collaborative teams, using a selection of electronic resources such as instruments, databases and computational tools to approach scientific problems. Those teams working on Grid projects are keen to encourage biologists to get involved in the development process.

### $C_2M$ – configureable chemical middleware

Paul van der Vet discusses the growing need for ways to combine distributed resources such as databases and programs in modern science, which is becoming common in genomics projects. One solution is middleware (systems that write converters, or wrappers), which allows a researcher to set up a configuration of disparate resources to perform a given task. $C_2M$ is a configureable chemical middleware, initially designed to write converters for plaintext files with molecular structure information.

### Meeting Highlights: CSHL and Wellcome Trust joint meeting – Genome Informatics

Wixon *et al.* report on the first joint Cold Spring Harbor Laboratory and Wellcome Trust meeting on Genome Informatics, which was held on the Genome Campus, near Cambridge, UK. The meeting included sessions on Annotation Pipelines, Genome Assembly, Gene Prediction, Functional Genomics, Curation and Ontologies, and Genome Visualisation.

### Meeting Highlights: 15th International Mouse Genome Conference

We bring you a report on selected sessions and plenary lectures from the mouse genome conference held in Edinburgh from 21–24 October. We cover talks from the sessions on Genome sequencing and comparative analysis, Functional genomics, Informatics, and Mutagenesis.

### Website Review: pathway databases

Following on from the profile of web resources for protein-protein interactions in our last issue, we present a survey of pathway databases. The resources in this area are concerned with metabolic pathways and/or regulatory pathways. Some of these resources offer metabolic reconstruction based on competed genome sequence data. This area has great potential to evolve towards a more complex understanding of the networks in operation inside cells, sometimes referred to as systems (or integrative) biology.

Published online: 14 November 2001
Copyright © 2001 John Wiley & Sons, Ltd.

### Conference Calendar

In this issue we have our first instalment of our Conference Calendar, which provides a categorised and chronological listing of relevant conferences. Here we cover meetings scheduled to occur between November 2001 and February 2002.

The Genomics Website at Wiley www.wiley.co.uk/genomics

The Genomics website at Wiley is a DYNAMIC resource for the genomics community, offering FREE special feature articles and new information EACH MONTH.

Find out more about our new journals *Comparative and Functional Genomics,* and *Proteomics.*

Visit the Library for hot books in Genomics, Bioinformatics, Molecular Genetics and more.

Click on Journals for information on all our up-to-the minute journals, including: *Genesis, Bioessays, Journal of Mass Spectrometry, Gene Function and Disease, Human Mutation, Genes, Chromosomes and Cancer* and the *Journal of Gene Medicine.*

Let the Genomics website at Wiley be your guide to genomics-related web sites, manufacturers and suppliers, and a calendar of conferences.

WILEY

2380

Copyright © 2001 John Wiley & Sons, Ltd. 

